# New ECG Compression Method for Portable ECG Monitoring System Merged with Binary Convolutional Auto-Encoder and Residual Error Compensation

**DOI:** 10.3390/bios12070524

**Published:** 2022-07-14

**Authors:** Jiguang Shi, Fei Wang, Moran Qin, Aiyun Chen, Wenhan Liu, Jin He, Hao Wang, Sheng Chang, Qijun Huang

**Affiliations:** School of Physics and Technology, Wuhan University, Wuhan 430072, China; shijig@whu.edu.cn (J.S.); ml_wangfei@whu.edu.cn (F.W.); qinmrmr@whu.edu.cn (M.Q.); aiyunch@whu.edu.cn (A.C.); whliu@whu.edu.cn (W.L.); jin.he@whu.edu.cn (J.H.); wanghao@whu.edu.cn (H.W.); changsheng@whu.edu.cn (S.C.)

**Keywords:** binary convolutional auto-encoder (BCAE), residual error compensation (REC), electrocardiogram (ECG), signal compression, portable ECG monitoring system

## Abstract

In the past few years, deep learning-based electrocardiogram (ECG) compression methods have achieved high-ratio compression by reducing hidden nodes. However, this reduction can result in severe information loss, which will lead to poor quality of the reconstructed signal. To overcome this problem, a novel quality-guaranteed ECG compression method based on a binary convolutional auto-encoder (BCAE) equipped with residual error compensation (REC) was proposed. In traditional compression methods, ECG signals are compressed into floating-point numbers. BCAE directly compresses the ECG signal into binary codes rather than floating-point numbers, whereas binary codes take up fewer bits than floating-point numbers. Compared with the traditional floating-point number compression method, the hidden nodes of the BCAE network can be artificially increased without reducing the compression ratio, and as many hidden nodes as possible can ensure the quality of the reconstructed signal. Furthermore, a novel optimization method named REC was developed. It was used to compensate for the residual between the ECG signal output by BCAE and the original signal. Complemented with the residual error, the restoration of the compression signal was improved, so the reconstructed signal was closer to the original signal. Control experiments were conducted to verify the effectiveness of this novel method. Validated by the MIT-BIH database, the compression ratio was 117.33 and the root mean square difference (PRD) was 7.76%. Furthermore, a portable compression device was designed based on the proposed algorithm using Raspberry Pi. It indicated that this method has attractive prospects in telemedicine and portable ECG monitoring systems.

## 1. Introduction

Electrocardiogram (ECG) is a bioelectrical signal test which provides information about human heart activity [1]. It is widely used by medical institutions because it is non-invasive and inexpensive. With the rapid development of wearable ECG detection systems and telemedicine applications in healthcare [2], ECG signals generated by these devices need to be stored and transmitted. However, 12-lead ECG signals require large storage space [3]. For example, an hour-long ECG record with a sampling rate of 360 Hz and a data resolution of 11 bits per sample has a size of 20.39 megabytes. Long-time ECG signal detection, such as with a Holter monitor, will create a very large amount of data. 

Cardiovascular diseases are mainly monitored by ECG, but the application of traditional ECG equipment is limited to professional medical institutions. In order to achieve more comprehensive monitoring of patients (such as community monitoring, home monitoring, etc.), there is an increasing demand for portable ECG monitoring systems. Generally, portable ECG monitoring systems use wireless technology to transmit ECG data, which is inconvenient for real-time transmission of large amounts of ECG data. Fortunately, signal compression technology can solve this problem by compressing the ECG signal before data transmission, which reduces the amount of transmitted data while ensuring the effect of diagnosis and treatment. Therefore, it is critical to choose an efficient compression coding technology. The traditional lossless compression method has a small compression ratio, which makes it difficult to meet the real-time data transmission requirements. The near-lossless compression method can achieve a high compression ratio and low signal distortion at the same time, meeting the requirements of portable ECG monitoring systems [4]. In the following experiments, the effectiveness of our near-lossless compression method and reconstruction scheme was verified. Generally, there are two aspects to achieving near-lossless ECG compression: transform-based methods and deep learning methods [5]. 

Transform-based methods mainly convert the signals into transform domain and abandon information which is not helpful for signal reconstruction. Due to the energy compaction property, Fourier transform (FT), wavelet transformation (WT), and discrete cosine transform (DCT) have shown validity in ECG compression [6]. By encoding the critical information, the ECG signal can be compressed. In ref. [7], P. Ziran et al. extracted frequency information by lifting wavelet transformation and discarding the insignificant information. The Embedded Zerotree Wavelet (EZW) was used to select features and improve the compression ratio. In ref. [8], Chunyu Tan presented an adaptive Fourier decomposition (AFD) with application to ECG compression. It sped up the de-composition and improved compression performance. In ref. [9], JiaLi Ma et al. fused AFD with the symbol substitution (SS) technique. AFD guarantees high fidelity and SS improves the compression rate without information loss. In ref. [10], Sibasankar Padhy et al. presented a compression method on multi-lead ECG records by using singular value decomposition in the multiresolution domain. In ref. [11], M.L. Hilton introduced wavelet transform in ECG compression. By combining it with EZW coding, the ECG signal can be compressed. However, there are two disadvantages to these transform-based methods. Firstly, these methods reduce the signal size by discarding some parameters directly, but some critical information is carried by these parameters, so these processes will degrade compression quality. Secondly, transform-based methods are always combined with the independent encoding algorithm, which will also increase computing complexity [12]. Therefore, transform-domain-based methods are not suitable for application in portable systems.

Recently, deep learning compression methods have become more popular for their high-quality compression, and the above two problems do not occur in the deep learning compression method. According to Andrew Y. Ng, DNNs can recognize patterns and learn useful features from raw input data without requiring extensive data preprocessing, feature engineering, or handcrafted rules, making them particularly suitable for interpreting ECG data [13]. As an end-to-end method, deep learning based on an auto-encoder can directly compress the ECG signal without additional encoding algorithms. Auto-encoder is a promising technique used in obtaining the low-dimensional representation of original signal and information restoration [14,15,16,17], which is a classical end-to-end deep learning algorithm. In ref. [18], Ozal Yildirim implemented a deep convolutional auto-encoder in the compression of ECG signals. A model of 27 stacked layers guarantees the quality of compression. In ref. [19], Wang et al. presented a spindle structure of a convolutional auto-encoder to increase the sufficient information extraction and the compression ratio. All these ECG compression methods based on deep learning rely on reducing hidden nodes to increase the compression ratio. However, the reduction of nodes in the hidden layer will degrade the quality of reconstruction [20]. In the above articles, the implementation of high ratio compression inevitably sacrifices the quality of reconstruction. However, portable ECG detection systems need to ensure good signal quality, so it is necessary to maintain the compressed signal quality while achieving a high compression ratio.

In this paper, a novel deep learning compression method was presented, which is based on binary convolutional auto-encoder (BCAE) equipped with residual error complement (REC). In this method, the convolutional auto-encoder (CAE) was determined as the base model [21]. CAE encoder encodes the input signal to obtain the compressed code of floating-point type, and then the CAE decoder decodes the compressed code to obtain the reconstructed signal. The novelty of BCAE is the binary output of the encoder section. By altering the activation function and gradient, the encoder can directly generate a binary code without extra coding. In this way, the floating nodes of CAE can be replaced by binary nodes. Without reducing the compression ratio, BCAE can greatly increase hidden nodes to improve the restoration capability of the network. Moreover, to further improve the compression quality, a new optimization model named residual error compensation (REC) was developed. It is a network to obtain the residual error between the output of BCAE and the original signals. Compensated with this residual error, the reconstructed signal can be more similar to the original signal. Thus, the novel strategy of BCAE + REC is an attractive method in both high reconstruction quality and high compression ratio. 

Compared with previous compression methods, the innovations of the method proposed in this paper are the following:

1. BCAE directly generated binary compressed code. Under the premise of a high compression ratio, hidden nodes were increased to improve the reconstruction quality.
2. By using REC, the quality of the reconstructed signal from BCAE was improved, which guarantees the compression quality.
3. Five categories of signals (normal beat, left bundle branch block beat, right bundle branch block beat, atrial premature beat, and premature ventricular contraction) from the MIT-BIH database were classified using the original and reconstructed signals, respectively, further verifying the effectiveness of the compression.
4. A portable device based on Raspberry Pi was designed to realize the proposed compression algorithm. It was proven that BCAE has practicality and is helpful for the application of portable ECG monitoring systems.

In summary, the ECG compression method proposed in this paper has a high compression ratio and little signal distortion, and so can be used for the transmission and storage of ECG data. The experiments verified that the proposed scheme can meet the requirements of portable ECG monitoring systems for data transmission while ensuring the effect of diagnosis and treatment.

The rest of this paper is organized as followings. Section 2 introduces the datasets used in the model and principle of proposed BCAE and REC, followed by model configuration. Section 3 introduces the evaluation criteria and shows detailed results. Section 4 presents the discussion and comparison. Finally, Section 5 concludes this paper.

## 2. Materials and Methods

This section first introduces the MIT-BIH database and ECG signal preprocessing, then explains the principle of the proposed method, and finally illustrates the model configuration. 

**Figure 1:**
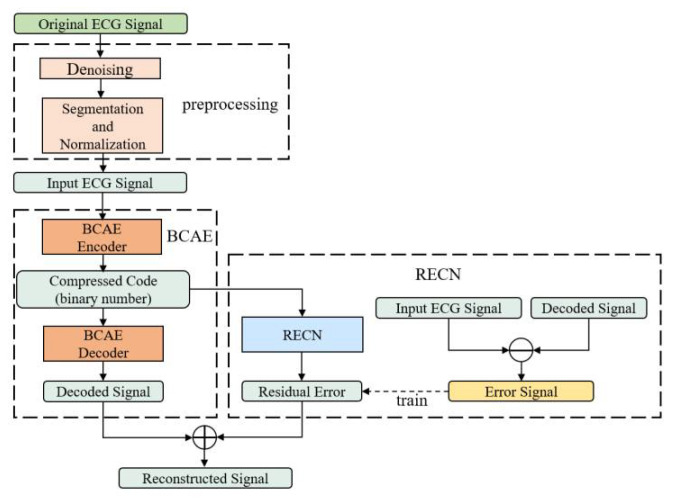
Structure of the proposed ECG compression method.

As shown in Figure 1, the method proposed in this paper contains three parts: ECG raw signal preprocessing, the binary convolutional auto-encoder (BCAE), and the residual error compensation network (RECN).

**Figure 2:**
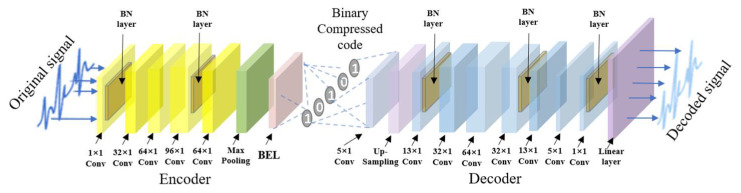
Sketch map of the structure of BCAE.

**Figure 3:**
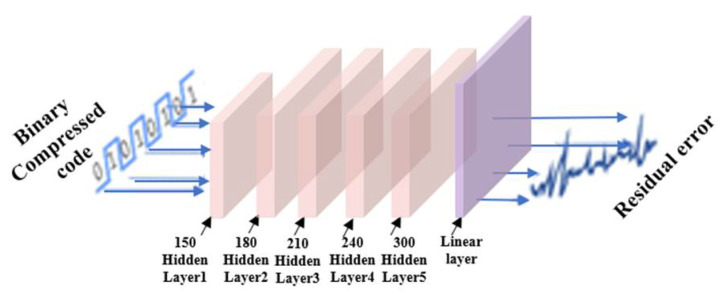
Sketch map of the structure of RECN (MLP).

The structures of BCAE and RECN are shown in Figure 2 and Figure 3. The first advantage is the BCAE which can generate the binary compressed output by encoding the hidden features. As shown in Figure 2, the encoder is composed of six 1-D convolutional layers to extract information as the feature vectors in the hidden layers. Through the binary convolutional layer, these features can be encoded into the binary codes. In this way, a conventional floating node can be replaced by a series of binary nodes. With nodes increasing, the effect of signal reconstruction is improved, ensuring a high compression ratio. These binary codes can be restored to the original signal by stacked deconvolutional layers in the decoder. The second advantage is the REC network which can compensate the loss to improve the reconstruction performance. As depicted in Figure 1 and Figure 3, RECN is designed to reduce the residual between the input ECG signal of the BCAE Encoder and the decoded signal of the BCAE Decoder output. Combined with the output of RECN, the reconstructed signal by BCAE can be higher quality. Details of each part are introduced as follows.

### 2.1. Datasets

The MIT-BIH database is provided by the Massachusetts Institute of Technology. It is one of the three standard ECG databases in the world and has been widely used to train the proposed network [22]. It contains 48 records from 47 patients, each with the diagnosis of several cardiologists. In the database, all signals are sampled at a frequency of 360 Hz with a resolution of 11 bits.

Raw ECG carries redundant information such as noises and low-energy components. In preprocessing stage, the noise of ECG signals was removed by a 0.5–150 Hz bandpass filter. Therefore, eliminating these redundancies is good to retain important information for compressing the ECG signals. Furthermore, all signals were normalized by the max–min normalization technique [23]. Deep learning compressed methods usually process on beats. Like many previous deep learning compressed methods, single beats were used as basic samples. This requires heartbeat segmentation of the original records. In the heartbeat segmentation stage, R-peak detection was first performed on each ECG recording using the Pan–Tompkins algorithm [24]. Then, 127 sample points on the left side and 192 sample points on the right side of the R peak were taken to obtain a heartbeat containing 320 (127 + 192 + 1 = 320) points [25]. At this point, the samples containing 320 11-bit floating-point numbers required for the training phase had been obtained. 

### 2.2. Binary Convolutional Auto-Encoder (BCAE)

**Figure 4:**
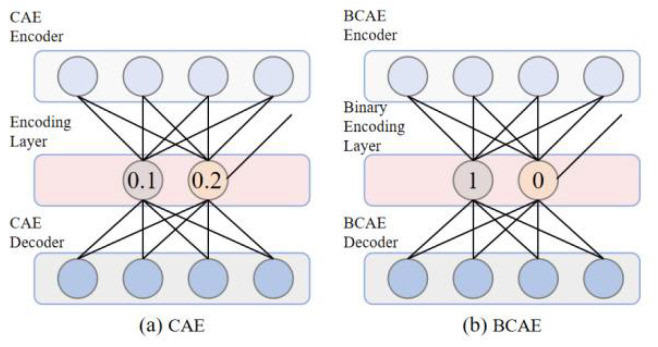
The compression method: (**a**) conventional CAE. (**b**) BCAE.

The proposed BCAE was developed from the traditional convolutional auto-encoder (CAE) [26]. CAE is an available technique successfully used in ECG compression. Considering its acceptable compression ability, CAE was used here as a basic model. However, conventional CAE achieves a high compression ratio by reducing the number of floating hidden nodes. Similar to CAE, BCAE can be also separated into encoder and decoder parts. In the encoder, convolution layers and pooling layers extract feature vectors with critical information from input signals. The improvement of BCAE is the binary encoding layer of the encoder, which replaces the conventional floating-point output with binary codes. For example, as shown in Figure 4a, the traditional CAE compresses the original signal into floating-point numbers; two 11-bit floating-point numbers occupy 22 bits, while in Figure 4b, BCAE compresses the ECG signal into binary numbers, and two binary numbers occupy only 2 bits. Therefore, compared with the traditional floating-point compression method, even if the number of hidden nodes of BCAE increases by 11 times, the compression ratio will not decrease. Enough numbers of hidden nodes can guarantee the reconstruction quality of the network and improve the compression quality [20]. Therefore, BCAE has great potential to achieve high compression performance. After training, transposed convolutional layers and up-sampling layers of the decoder help rebuild original signals from binary code.

The detailed operations of the binary encoding layer (BEL) and function layers used in the proposed model are illustrated in the following sections.

#### 2.2.1. Binary Encoding Layer

The improvement is to modify the activation function and gradient of the convolutional layer to generate the binary output. The binary technique has been employed in the convolutional network (CNN) successfully [27]. In our work, the most important modification is to utilize the step function as the activation function *f* (•):

(1) $$f\left(x\right)=\left\{\begin{array}{cc} 0, & x<0\\ 1, & x\geq 0 \end{array}\right.$$

**Figure 5:**
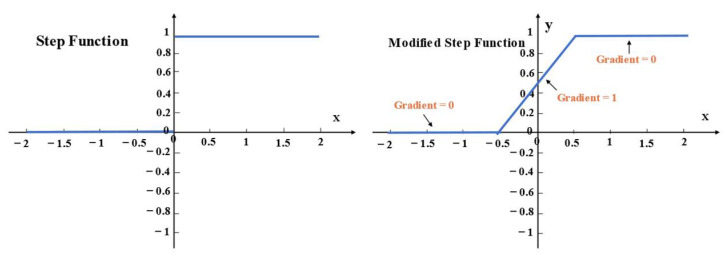
Step function and modified step function.

Due to the binary output of 0 and 1, this layer can directly achieve binary encoding. Thus, a 20-bit floating node in conventional CAE can be replaced by 20 binary nodes in BCAE. Under the same compression ratio, BCAE has more nodes. With nodes increasing, the quality of the reconstructed signal can be improved to guarantee the compression quality. This work used the backpropagation algorithm to train the network, which requires the input to be differentiable at every point. However, the above step function is not differentiable at *x* = 0. Therefore, in this work, the gradient in a small range near zero was modified to a constant 1. In Figure 5, from −0.5 to 0.5, the gradient *g* (•) as Formula (2) calculated is 1 and the other gradients are 0.

(2) $$g\left(x\right)=\left\{\begin{array}{cc} 0, & x<-0.5\\ 1, & -0.5\leq x<0.5\\ 0, & x\leq 0.5 \end{array}\right.$$

#### 2.2.2. 1-D Convolutional Layers

These layers use the convolution kernel to perform convolution operations on the input data and output the result through the activation function. The kernels are a series of filters with trainable weights. Through training, kernels can extract the significant information from the input and discard the redundancy. The convolution operation can be described by Formula (3).

(3) $${x_{conv}}_{j}^{l}=f\left(\sum_{i\in M_{j}}x_{i}^{l-1}\times \omega_{ij}^{l}+b_{j}^{l}\right)$$

in which ${x_{conv}}_{j}^{l}$ denotes the jth output of the lth convolution layer, $\omega_{ij}^{l}$ is the weight between ${x_{conv}}_{j}^{l}$ and $x_{i}^{l-1}$; $M_{j}$ represents the connection between $x_{j}^{l-1}$ and ${x_{conv}}_{j}^{l}$; ∗ is the 1D convolution operation; $b_{j}^{l}$ is the bias of jth output from the lth convolution layer. Additionally, $f\left(•\right)$ represents the activation function. In this paper, the hyperbolic tangent function (tanh) was used as an activation function in all convolutional layers except the binary encoding layer.

#### 2.2.3. Transposed Convolution Layer

These layers achieve signal restoration by deconvolution. As an inverse process of convolution, deconvolution is to convolve the transposed 1-D kernels with input signals as Formula (4) defines:

(4) $${x_{Tconv}}_{j}^{l}=f\left(\sum_{i\in M_{j}}x_{i}^{l-1}\times {\left(\omega_{ij}^{l}\right)}^{T}+b_{j}^{l}\right)$$

where ${x_{Tconv}}_{j}^{l}$ is the jth output of the lth Transposed Convolution layer, and T represents the transpose operation. The other parameters have the same meaning as the 1-D convolutional layer. By stacked transposed convolution layers, the compressed code can be restored to the original signal.

#### 2.2.4. Max Pooling Layer

The max pooling layer is always adopted to reduce the dimension of features by down-sampling. As Formula (5) defines, max pooling retains the maximum value in the range of the pooling window and discards other values while moving this window.

(5) $${x_{Mpool}}_{j}^{l}\left(n\right)=\underset{r\in R}{max}\left[x_{j}^{l-1}\left(n\times S+r\right)\right]$$

in which ${x_{Mpool}}_{j}^{l}\left(n\right)$ is the nth value of jth output from lth max-pooling layer; R represents the size of the window; and S is the stride pooling layer. R was set to be equal to S for a nonoverlapped pooling. r is the index of the sampling window. The information can be centralized for better compression by this layer. Moreover, it can alleviate the computational burden and avoid overfitting. 

#### 2.2.5. Up-Sampling Layer

Up-sampling is commonly used in feature extensions. Here, zero-padding is used instead of interpolation to reduce computational complexity. In each up-sampling window, the first value is restored by the corresponding input and the rest is padded with zeros. This operation is formulated as Formula (6):

(6) $$\left\{\begin{array}{c} {x_{Us}}_{j}^{l}\left(n\times S^{\prime }+r\right)=x_{j}^{l-1}\left(n\right)\;\;\;\;\;\;\;\;\;\;\;\;\;\;\;\;\;\;r=1\\ {x_{Us}}_{j}^{l}\left(n\times S^{\prime }+r\right)=0\;\;\;\;\;\;\;\;\;\;\;\;\;\;\;\;\;\;\;r=2,\;3\ldots R^{\prime } \end{array}\right.$$

where $R^{\prime }$ represents the size of the up-sampling window and $S^{\prime }$ is the stride of sampling; the other parameters in Formula (6) are as same as those in the encoder. In this way, compressed data can be restored to the original size.

#### 2.2.6. Linear Layer

The ECG signal is reconstructed from features by linear transform in this layer. Because of the hyperbolic tangent activation, the output of the transposed convolutional layer is limited between −1 and 1. To address this problem, a linear layer was used to rebuild the original ECG signals. The linear layer operates as:

(7) $${x_{Linear}}_{j}^{l}=\sum_{i\in M_{j}}x_{i}^{l-1}\times {\left(\omega_{ij}^{l}\right)}^{T}+b_{j}^{l}$$

where ${x_{Linear}}_{j}^{l}$ is the jth output in lth linear layer; $\omega_{ij}^{l}$ and $b_{j}^{l}$ have the same definition as that in Formula (4).

As depicted in Figure 2, these function layers are stacked to generate a BCAE network. The encoder of BCAE consists of stacked convolutional layers, max-pooling layers, and a binary encoding layer. The first few convolutional layers and max-pooling layers extract the main features and condense the features. The last of the encoder is the binary coding layer, which directly compresses the signal into binary code. As for the decoder of BCAE, it consists of eight transposed convolution layers, up-sampling layers, and a linear layer. The binary compressed code can be restored to the original signal by transposed convolution layers and up-sampling layer. The linear layer further transforms the feature signal into the reconstructed signal with accurate amplitude.

To strengthen the performance of BCAE, Batch Normalization (BN) and dropout are introduced after 1-D convolutions and transposed convolutions for reducing overfitting [28]. As a deep learning model, BCAE can be also improved by BN, which eliminates the gradient vanishing and speeds up the convergence in the training phase [29]. Furthermore, as an effective technique to avoid overfitting, dropout is also employed to improve the compression performance [30]. In summary, all these strategies make BCAE a promising model in ECG signal compression.

### 2.3. Residual Error Compensation Network (RECN)

RECN can be treated as a complement of BCAE. It is designed to obtain the residual error between the reconstructed signal by BCAE and the original signal. The main structure of RECN is depicted in Figure 3. The binary compressed code was used as the input of RECN. A Multi-Layer Perceptron (MLP) was set to transform the binary compressed data to the desired residual error. After BCAE training, the reconstructed signal and corresponding binary code could be obtained. Through the original signal, the difference between the original signal and the reconstructed signal could be obtained, which is the label of the RECN training process. The RECN network was trained based on this label to make the reconstructed signal closer to the original signal. Because the corresponding residual error of the reconstructed signal is actually small, the weight update speed is very slow. Therefore, the residual error was magnified ten times as the output of RECN for more efficient training. Therefore, the output of RECN is reduced by ten times before it is added to the output of BCAE. In summary, as an optimization method, RECN can compensate for the output of BCAE by outputting residual error. In this way, optimized by RECN, the compression quality of BCAE can be further improved.

### 2.4. Compression Package

**Figure 6:**
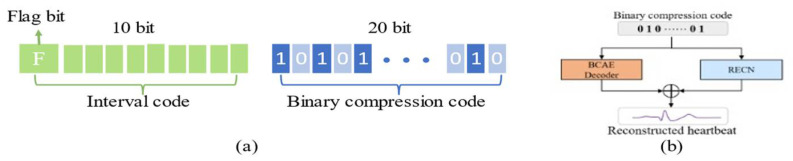
(**a**) Structure of the compression package. (**b**) Process of obtaining the reconstructed signal.

As shown in Figure 6a, the compression process of the heartbeat signal contains two parts: interval code and binary compression code. The interval code stores the interval between the current beat and the previous beat, represented by a 10-bit binary code [19]. The first bit of the interval code is the flag bit. When it is set to 0, the beat needs to be delayed by the corresponding time to connect with the previous beat, and interpolation is used to pad the delay interval. When the flag bit is set to 1, the beat should be connected to the previous beat by the corresponding time earlier, and the overlapping part is represented by an average value of the overlapping signals. The remaining 9 bits represent the delay interval or the duration of the overlapping part, which can represent the time interval within 512 (2^9^ = 512) sampling points (512 samples/360 Hz = 1.4 s). Considering that the heartbeat duration is usually not more than 1.4 s, through the above connection method, the reconstructed heartbeats segment can be restored to the original ECG signal by using this 10-bit interval code. As for binary compressed code, it is generated by BCAE. According to the structural configuration of BCAE, the output nodes of the encoder are 20, so the size of the compression code is fixed. Therefore, the compressed heartbeat can be encoded into a 20-bit binary code. In this way, a heartbeat with 320 samples can be compressed into a 30-bit binary compression pack.

As shown in Figure 6b, in the reconstruction stage, the BCAE decoder and RECN decode the binary compression code to obtain the reconstructed heartbeat (waveform for floating point). Since the interval information of adjacent heartbeats is stored in the interval code, continuous ECG records can be obtained through the interval code.

### 2.5. Model Configuration

The proposed architecture and parameter configuration are summarized in Table 1 and Table 2. As for BCAE, the encoder consists of 9 convolutional layers, 4 max-pooling layers, and 1 binary encoding layer. The decoder consists of 9 transposed convolutional layers, 4 up-sampling layers, and 1 linear layer. As for RECN, it consists of 5 hidden layers and 1 linear layer. Here, all convolutions and transposed convolutions were operated as the “same” pattern, which pads the output to the input size.

Tensorflow [31] (Python version) was used to build and train the proposed network. Since the noises have the same frequency as ECG signals and cannot be removed [4], the Pseudo–Huber loss function was determined as the loss function, and defined as [32]:

(8) $$L_{\delta }\left(y\right)=\delta^{2}\left(\sqrt{1+{\left[\left(y-y^{\prime }\right)/\delta \right]}^{2}}-1\right)$$

where L represents the loss; y represents the original signal; and $y^{\prime }$ denotes the reconstructed signal. δ represents the parameter controlling the gradient less steep for extremums and was set to 0.9 after debugging. As a smooth approximation of the Huber loss, it guarantees derivation of each order [33]. To speed up the model convergence, the Adagrad optimizer [34] was used for training. The initial learning rate and batch size were set to 0.1 and 256, respectively. The number of training iterations was set to 400 and the model can converge.

## 3. Results

This section first introduces the evaluation criteria and then illustrates the experimental results of ECG compression on the MIT-BIH database.

### 3.1. Performance Evaluation

There are several metrics used for performance evaluation, which can be divided into two main aspects: reconstruction quality and compression efficiency. These performance criteria are summarized as compression ratio (*CR*), signal-to-noise ratio (*SNR*), root mean square error (*RMS*), percentage *RMS* difference (*PRD*), normalized version of *PRD* (*PRDN*), and quality score (*QS*) [4]. They are defined as follows:

(9) $$CR=\frac{c_{i}}{c_{o}}$$

(10) $$SNR=10\times \mathrm{lg}[\frac{\sum_{i=1}^{\;n}{\left(D_{o}\left(i\right)-D_{m}\right)}^{2}}{\sum_{i=1}^{\;n}{\left(D_{o}\left(i\right)-D_{r}\left(i\right)\right)}^{2}}]$$

(11) $$RMS=\sqrt{\frac{\sum_{i=1}^{\;n}{\left(D_{o}\left(i\right)-D_{r}\left(i\right)\right)}^{2}}{L}}$$

(12) $$PRD\left(\%\right)=\sqrt{\frac{\sum_{i=1}^{\;n}{\left(D_{o}\left(i\right)-D_{r}\left(i\right)\right)}^{2}}{\sum_{i=1}^{i=n}{\left(D_{o}\left(i\right)\right)}^{2}}}\times 100$$

(13) $$PRDN\left(\%\right)=\sqrt{\frac{\sum_{i=1}^{\;n}{\left(D_{o}\left(i\right)-D_{r}\left(i\right)\right)}^{2}}{\sum_{i=1}^{n}{\left(D_{o}\left(i\right)-D_{m}\right)}^{2}}}\times 100$$

(14) $$QS=\frac{CR}{PRD}$$

where $c_{i}$ and $c_{o}$ represent the size of the input signal and compressed signal, respectively. $D_{o}$, $D_{r}$ are original signal, reconstructed signal and $D_{m}$ is the mean value of the original signal. L denotes the length of the signal. *CR* is widely accepted as a criterion of compression efficiency. As for reconstruction quality evaluation, *RMS* and *PRD* are proportional to the difference between the original signal and the reconstructed signal. Moreover, *SNR* is used to evaluate the magnitude of the real signal and background noise. To evaluate the performance comprehensively, *QS* was used to indicate the comprehensive performance of both compression efficiency and reconstruction quality [35]. The smaller the *PRD*, the better the quality of the reconstructed signal. The larger the compression ratio, the higher the compression efficiency, but the quality of reconstruction tends to be worse. *QS* is the ratio of *CR* and *PRD*, so the larger the *QS*, the better the overall quality of compression.

### 3.2. Experimental Results

**Figure 7:**
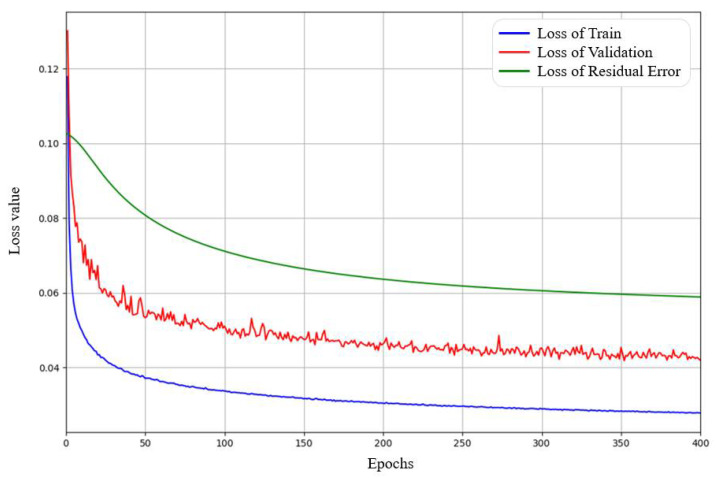
Graph of variation of the loss value versus epochs for BCAE and RECN.

The MIT-BIH database has 48 records, and 1000 heartbeats were randomly selected from each record for research. These 48,000 beats were divided into the training set, validation set, and test set. The model was trained on 38,400 beats, containing 80% of the data. The optimizations were performed on 7200 validation beats. The remaining 2400 beat samples were used to evaluate performance in the testing phase, which contains 50 heartbeats from each record. The model was trained for 400 epochs with a batch size of 256, and the training time per epoch was about 10 s. As shown in Figure 7, the loss of BCAE and RECN in the training phase can be visually analyzed. It can be demonstrated that both BCAE and RECN converged to a low loss. The training losses of BCAE and RECN decreased from 0.118 to 0.027 and 0.102 to 0.059, respectively. As for validation losses, they decreased synchronously, which indicates that the model is not overfitting.

After training, performance evaluation was conducted on the network with the aforementioned performance criteria in Section 3.1. To further evaluate the performance of the proposed method on each record, the trained model was tested on all 48 records. Finally, the results are summarized in Table 3. The CR is related to the network’s structure rather than the input signal. Since the input heartbeat contains 320 11-bit floating-point numbers, and the compressed package is a 30-bit binary number, CR is 117.33 (320 × 11 bit/30 bit) on each record.

Table 3 shows that the proposed model achieved good compression performance with a low PRD of 7.76 and RMS of 0.026 respectively. Moreover, the average SNR maintained at 15.93 dB. The high CR and low PRD indicated that the proposed method performs an effective compression. To examine the performance of each record, the lowest PRD value at 2.59 demonstrated the best compression performance on record 207. In contrast, record 222 generated the highest PRD with the value of 22.86, which can be considered the worst case among all records.

**Figure 8:**
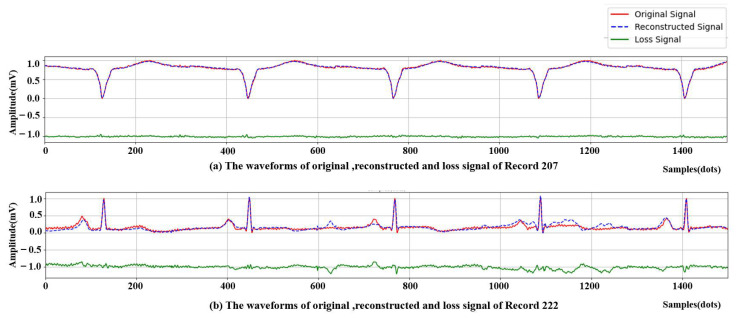
The comparison of the compression performance. (**a**) The best compression quality: Record 207. (**b**) The worst compression quality: record 222. For ease of viewing, the loss signal is shifted down by one unit to the −1 position.

To visually compare the performance, the reconstructed signals of records 207 and 222 by the proposed method are given in Figure 8. Notably, the network training is based on beats, so the reconstructed signals were concatenated by reconstructed single beats. To obtain the complete reconstructed ECG signal, the interval information between two adjacent heartbeats was converted into binary interval code in the preprocessing process. Through these binary interval codes and the compressed code of a single heartbeat, a continuous reconstructed ECG signal can be obtained. The Figure shows the comparison between the original signals and reconstructed signals. Their difference is denoted as the loss signal. For ease of viewing, the loss signal is shifted down by one unit to the −1 position. It can be seen from Figure 8a that the reconstructed signal in the best case is of high quality and has significant consistency with the original signal, and the signal type in the figure is arrhythmia, which proves the effectiveness of compression and reconstruction for different types of heartbeats. As for the worst case in Figure 8b, although there are some differences between the reconstructed signal and the original signal, they still reserve morphologies. Thus, the model can restore the morphology information successfully, even though some losses exist. In summary, our results verify the high reconstruction quality of the proposed method.

Since the input of BCAE is single heartbeat, it is necessary to evaluate the effect of changes in rhythms and morphologies on compression quality. For this purpose, records 100, 117, and 119 were used because of their variable rhythms and morphologies [32]. Compression performance on these three records and their respective rhythms are summarized in Table 4. 

**Figure 9:**
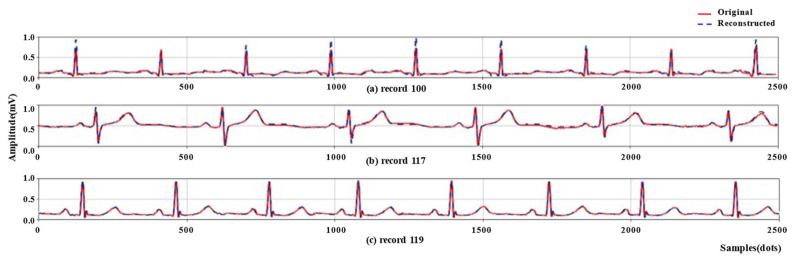
Compression performance of records 100, 117, and 119.

Figure 9 depicts the original signal and the reconstruction signal of 2500 sample points from records 100, 117, and 119. Due to the different rhythms, the number of heartbeats contained in 2500 sample points was also different. The best PRD was obtained on record 117 with a value of 3.75. It had a RR interval of 422.85 samples, larger than the input size. The worst case was generated by record 100 with PRD at 8.06, the RR interval was 286.05 samples, smaller than the input size. Comprehensively, under these different rhythms, the proposed method maintains the capacity of high-quality reconstruction with average PRD at 5.44, RMS at 0.020, and SNR at 17.48, respectively. As for different morphologies, the method proposed in this paper can reconstruct the binary codes into the original signal with high quality. The losses are acceptable and overall morphologies can be successfully obtained. Therefore, the proposed method can successfully deal with compression on ECG signals which have variable rhythms and morphologies.

## 4. Discussion

**Figure 10:**
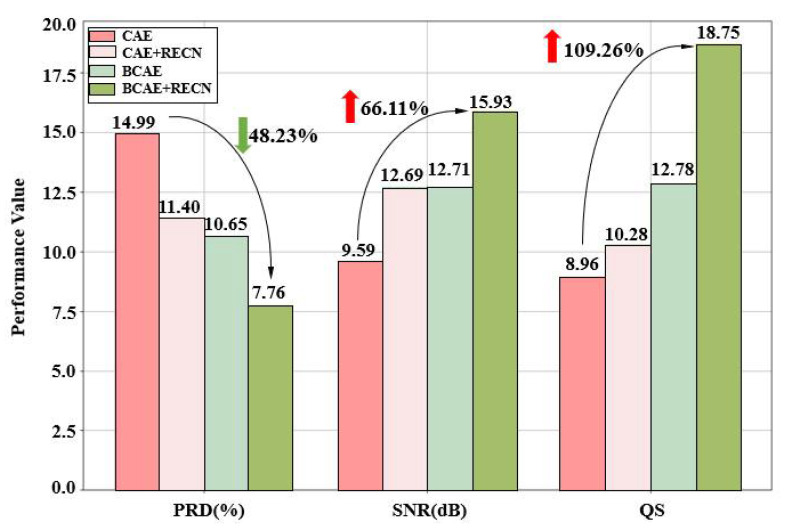
The performance improvement of BCAE and RECN. CR: 117.33.

More advantages of BCAE and RECN are illustrated in this section. Firstly, the improvements from BCAE and RECN are summarized in Figure 10. Four models, CAE, BCAE, CAE + RECN, and BCAE + RECN were tested with a generic test set of 2400 beats. The compression ratios of the four models are controlled by the hidden layer nodes to be equal, and the reconstruction quality results are represented by the histogram. It can be noted that BCAE was much better than CAE in compression quality under the same compression ratio. The PRD decreased to a low level of 10.65% and the SNR was improved to 12.71 dB. This result proves the effectiveness of BCAE on reconstruction quality. By the innovative binary compressed code, quality improvement can be achieved without sacrificing the compression ratio. 

Secondly, optimized with RECN, the compression quality was also further improved. It further reduced PRD to 7.76 and enhanced QS to 18.75. Complemented with the residual error, the reconstructed results can be more accurate. Hence, it was proved that the RECN, a novel optimization method proposed in this paper, boosts the compression quality. In all, the proposed BCAE compression model with RECN can achieve an attractive ECG compression.

In Table 5, the results of the method proposed in this paper are compared with several existing studies on ECG compression. The main comparison is the average result on the records. According to Table 5, though ref. [18] achieves lower PRD values, this is always due to the high offset line and different amplitude dimensions. To objectively evaluate the compression quality, PRDN was also evaluated, which removes the offset influence. The proposed method has good compression quality with a PRD of 7.76, and has the largest compression ratio of 117.33 and the highest QS of 18.75. QS is the evaluation metric that best represents the comprehensive compression effect, and high QS proves the advantages of the proposed method. Compared with refs. [7,36], and [37], with the approximate compression quality (PRD), the proposed BCAE strategy can greatly improve the CR and QS. In summary, the proposed method maintains a high quality and high ratio compression, achieves optimal overall performance, is attractive in ECG compression, and can be used for portable ECG monitoring systems.

**Figure 11:**
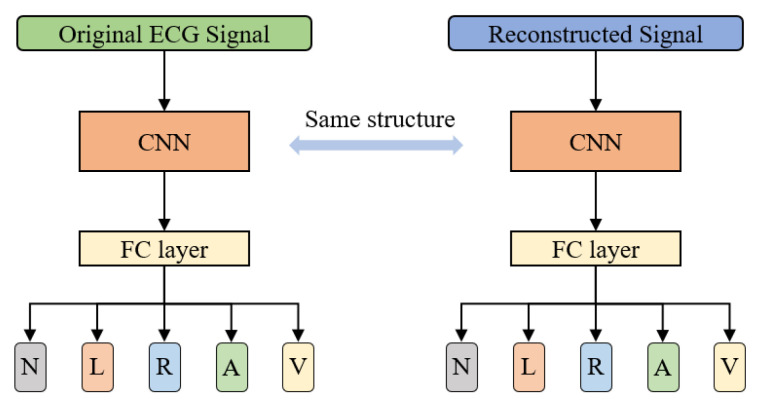
Arrhythmia detection using original and reconstructed signals separately.

To further verify the quality of the reconstructed signal, as shown in Figure 11, five types of beats: Normal beat (N), Left bundle branch block beat (L), Right bundle branch block beat (R), Atrial premature beat (A), and Premature ventricular contraction (V) were classified using the original signals and reconstructed signals, respectively. In order to be consistent with the number of heartbeats compressed and reconstructed in Section 3.2, a total of 48,000 heartbeats of the above five types in the MIT-BIH database were obtained according to the heartbeat extraction method and preprocessing method mentioned in Section 2.1. These beats were compressed and reconstructed using the method proposed in this paper. To reproduce the visual inspection performed by a cardiologist, this experiment analyzed the signals in the time domain. Here, a convolutional neural network (CNN) [39] is directly used to classify the heartbeat waveforms of the reconstructed and the original signals, respectively. Of the beats of each class, 80% were used as the training set and 20% were used as the test set. The classification results are shown in Table 6.

This experiment compares the classification results of the original signal and the reconstructed signal, rather than pursuing the classification effect. In order to reduce the influence of other features on the classification, this experiment only analyzed the waveform features, that is, only the time domain signal was used for classification. The results showed that the difference in accuracy and average F1 score for the five-class signal classification using the original and reconstructed signals is small (less than 1%), which is acceptable, demonstrating the effectiveness of the proposed compression method.

**Figure 12:**
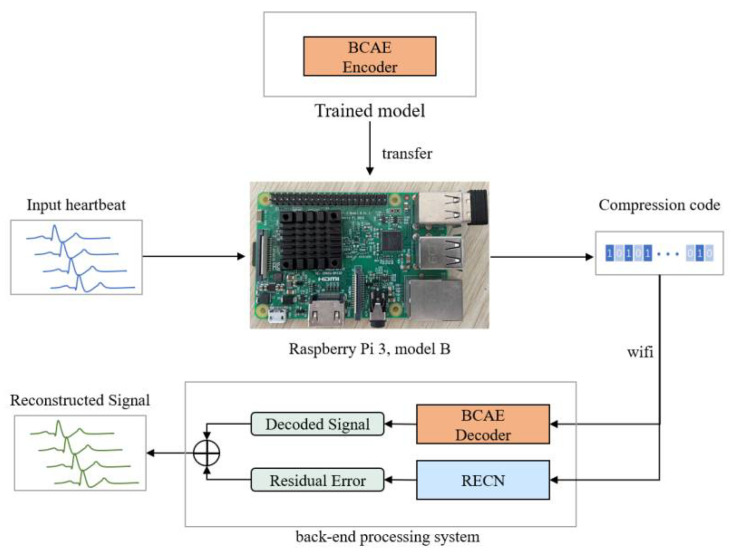
Structure of the portable ECG compression device.

In addition, to verify the practicability of the proposed method, a portable ECG signal compression device was made using Raspberry Pi 3 Model B (as shown in Figure 12). Transfer the neural network model (BCAE Encoder) trained in Section 3.2 into the Raspberry Pi and compress the 2400 heartbeats from the training set. The compression code is transmitted to the back-end processing system (such as a computer) through wifi, and the signal is reconstructed through the BCAE decoder and RECN. The reconstructed signal can be used for further disease detection. This experiment mainly calculates the time required for a single heartbeat containing 320 points to be compressed by the Raspberry Pi. The results are shown in Table 7.

According to the above experimental results, for a single heartbeat (with a duration of 320/368 HZ = 0.88 s), the compression processing time on the Raspberry Pi is 0.0101 s, which is much less than 0.88 s. This proves that the portable ECG signal compression device designed in this paper can realize real-time processing of ECG signals, so the proposed compression method has practical significance and can be used in wearable ECG devices.

## 5. Conclusions

In this paper, a novel ECG compression method of RECN and BCAE was proposed. The main objective of this study was to achieve efficient ECG signal compression through deep learning while ensuring the quality of the reconstructed signal and a high compression ratio. Based on the CAE model, the binary encoding strategy was introduced into BCAE, which can guarantee the compression ratio and improve the quality of reconstruction. BCAE was an end-to-end model that needs no extra encoding algorithm. Additionally, an efficient optimization technique, residual error compensation, was applied to improve the compression quality. Validated experimentally by the MIT-BIH database, the efficiency of the proposed method was proved. As a result, it achieves state-of-the-art performance with a compression ratio of 117.33 and PRD of 7.76. In addition, an experiment was designed to compare the classification results of the original heartbeat and the reconstructed heartbeat for Normal beat, Left bundle branch block beat, Right bundle branch block beat, Atrial premature beat, and Premature ventricular contraction. The differences in accuracy and average F1 scores were small, demonstrating that the ECG signals reconstructed by these codes were of high quality. Moreover, a portable compression device was designed based on the proposed compression algorithm using Raspberry Pi, which proves the practicality of the proposed method. In a summary, this method has attractive prospects in large data storage and portable electrocardiogram detection systems, and it can provide an effective compression method for remote data transmission, especially in portable ECG detection systems.

## Figures and Tables

**Table 1:** Detailed configuration information of BCAE.

Section	No.	Layer Name	Number of Filter×Kernel Size	Pooling (Up-Sampling) Stride/Size	Dropout	Activation Function	Output Size
Input		Original Signal					320×1
Convolutional Encoder	1	1D Conv	4×8	—	×	Tanh	320×4
	2	1D Conv + BN	8×32	—	×	Tanh	320×8
	3	Max Pooling	—	2	×	—	160×8
	4	1D Conv + BN	16×64	—	×	Tanh	160×16
	5	1D Conv + BN	32×96	—	×	Tanh	160×32
	6	Max Pooling	—	2	×	—	80×32
	7	1D Conv + BN	64×64	—	×	Tanh	80×64
	8	1D Conv + BN	64×32	—	×	Tanh	80×64
	9	Max Pooling	—	2	×	—	40×64
	10	1D Conv + BN	32×16	—	×	Tanh	40×32
	11	1D Conv + BN	16×8	—	×	Tanh	40×16
	12	Max Pooling	—	2	×	—	20×16
	13	1D Conv + BN	1×8	—	×	Tanh	20×1
	14	BEL	—	—	×	Step function	20×1
Compressed code (Input of decoder)		Binary Compressed Code					20×1
Convolutional Decoder	15	1D TConv	1×8	—	×	Tanh	20×1
	16	Up-sampling	—	2	×	—	40×1
	17	1D TConv + BN	16×8	—	√	Tanh	40×16
	18	1D TConv + BN	32×16	—	×	Tanh	40×32
	19	Up-sampling	—	2	×	—	80×32
	20	1D TConv + BN	64×32	—	×	Tanh	80×64
	21	1D TConv + BN	64×64	—	×	Tanh	80×64
	22	Up-sampling	—	2	×	—	160×64
	23	1D TConv + BN	32×96	—	√	Tanh	160×32
	24	1D TConv + BN	16×64	—	×	Tanh	160×16
	25	Up-sampling	—	2	×	—	320×16
	26	1D TConv + BN	8×32	—	×	Tanh	320×8
	27	1D TConv + BN	1×8	—	×	Tanh	320×1
	28	Linear layer	—	—	×	—	320×1
Output		Reconstructed Signal					320×1

BN: Batch Normalization. 1D TConv: 1D Transposed convolutional layer. BEL: Binary encoding layer.

**Table 2:** Detailed configuration information of RECN.

Section	No.	Layer Name	Activation Function	Output Size
Input	Binary Compressed Code			20×1
Hidden layers	1	Hidden layer 1	Relu	80×1
	2	Hidden layer 2	Relu	140×1
	3	Hidden layer 3	Relu	200×1
	4	Hidden layer 4	Relu	260×1
	5	Hidden layer 5	Relu	320×1
	6	Linear layer	—	320×1
Output	Residual Error			320×1

**Table 3:** Compression performance on the test set and each record in the MIT-BIH database.

Record	PRD(%)	PRDN(%)	RMS	SNR (dB)	QS	Record	PRD(%)	PRDN(%)	RMS	SNR (dB)	QS
100	8.06	14.41	0.016	17.07	14.56	202	15.06	20.90	0.032	14.00	7.79
101	10.89	14.18	0.018	17.31	10.77	203	13.43	29.78	0.053	11.17	8.74
102	9.37	22.65	0.027	13.07	12.52	205	11.14	19.72	0.023	14.75	10.53
103	8.44	14.12	0.017	17.68	13.91	207	2.59	12.47	0.021	18.49	45.35
104	6.26	20.41	0.025	14.08	18.74	208	11.47	21.91	0.042	13.72	10.23
105	11.13	15.89	0.025	16.43	10.55	209	6.59	24.32	0.025	12.50	17.81
106	11.28	23.30	0.037	13.27	10.41	210	9.82	18.23	0.031	15.78	11.94
107	4.92	15.77	0.027	16.35	23.84	212	8.93	19.49	0.029	14.50	13.14
108	6.36	20.19	0.031	14.25	18.45	213	5.90	17.06	0.027	15.78	19.89
109	4.26	9.11	0.015	21.05	27.54	214	6.92	11.10	0.018	19.52	16.96
111	8.69	21.16	0.031	13.77	13.5	215	5.83	18.14	0.028	15.08	20.11
112	5.10	17.12	0.021	15.45	23.00	217	4.61	15.48	0.027	16.44	25.44
113	6.80	12.11	0.017	18.75	17.25	219	7.69	13.03	0.019	17.85	15.27
114	5.26	24.84	0.037	12.35	22.29	220	4.22	16.81	0.016	16.08	27.80
115	3.39	11.07	0.011	19.31	34.56	221	9.63	17.11	0.033	15.84	12.19
116	6.99	15.96	0.019	16.79	16.80	222	22.86	40.88	0.047	8.47	5.13
117	3.75	16.39	0.022	16.29	31.27	223	8.38	16.35	0.026	16.89	14.00
118	3.02	10.40	0.014	19.77	38.80	228	8.28	20.15	0.038	14.72	14.17
119	4.51	11.67	0.021	19.07	26.04	230	3.50	15.16	0.016	16.71	33.57
121	11.80	14.53	0.026	17.03	9.94	231	4.37	14.41	0.016	16.95	26.82
122	8.72	12.34	0.02	18.57	13.46	232	7.19	21.61	0.027	13.50	16.32
123	4.22	13.22	0.014	17.86	27.82	233	4.19	13.99	0.025	17.51	28.00
124	8.12	11.52	0.019	19.24	14.44	234	15.09	18.82	0.025	14.75	7.77
200	4.22	17.19	0.032	15.74	27.82	Average of 48 records	7.76	17.53	0.026	15.93	18.75
201	9.31	24.98	0.044	12.91	12.60						

CR: 117.33 (320 × 11 bit/30 bit).

**Table 4:** Rhythm and compression performance on records 100, 117, and 119.

Record	PRD (%)	RMS	SNR (dB)	QS	Rhythm (Samples)
100	8.06	0.016	17.07	14.56	286.05
117	3.75	0.022	16.29	31.27	422.85
119	4.51	0.021	19.07	26.04	327.02
Average	5.44	0.020	17.48	23.95	319.11

Input size: 320 samples. CR: 117.33 (320 × 11bit/30bit). Rhythm: the average of the RR interval.

**Table 5:** Comparison with previous work.

No.	Year	Method	Data	Records	CR (Average)	PRD (%) (Average)	PRDN (%) (Average)	QS (Average)
1	2005	SPHIT [36]	MITdb	3 (100, 107, 119)	21.4	7.27	—	3.21
2	2016	PCA [38]	MITdb	All records	50.74	16.22	16.22	3.13
3	2017	EZW [7]	MITctdb	All records	9.27	8.17	—	1.13
4	2018	DCT [37]	MITdb	All records	6.27	5.37	7.95	1.49
5	2018	CAE [18]	MITdb	All records	32.25	2.73	31.17	11.81
7	2019	SCAE [19]	MITdb	All records	106.45	8.00	—	16.44
**Proposed Method**	**2022**	**BCAE + REC**	**MITdb**	All records	**117.33**	**7.76**	**17.53**	**18.75**

SPHIT: set partitioning in hierarchical trees coding. PCA: principal component analysis. EZW: embedded zerotree wavelet. DCT: discrete cosine transform composition. CAE: convolutional auto-encoder. SCAE: spindle convolutional auto-encoder. MITdb: MIT-BIH database. QS:CR/PRD. MITctdb: MIT-BIH ECG Compression Test Database. CR: compression ratio. PRD: percentage RMS difference. PRDN: normalized version of PRD. QS: quality score. (The four evaluation indicators are defined in Section 3.1).

**Table 6:** Classification results of original and reconstructed signals.

Signal Type	Accuracy	F1_Score (Average)	F1_Score				
			N	L	R	A	V
original signal	96.34%	93.05%	97.50%	98.50%	98.19%	79.23%	91.81%
reconstructed signal	95.77%	92.15%	97.13%	95.96%	97.42%	79.03%	91.18%

Accuracy = (TP + TN)/(TP + TN + FP + FN) F1_score = 2 × TP/(2 × TP + TN + FP + FN) TP: true positive; TN: true negative; FP: false positive; FN: false negative.

**Table 7:** Comparison of input signal time and signal processing time with Raspberry Pi.

	Time(s)
Input heartbeat	0.8889
Compression	0.0101

## Data Availability

MIT-BIH database is available at https://www.physionet.org/content/mitdb/1.0.0/ (accessed on 9 September 2021).
